# Supplementary material for: Effects of Walking Speed and Added Mass on Hip Joint Quasi-Stiffness in Healthy Young and Middle-Aged Adults
Source: Sensors (Basel). 2023 May 6;23(9):4517. doi: 10.3390/s23094517 (PMC10181717; doi:10.3390/s23094517)
Supplement: Supplementary file 1 [file sensors-23-04517-s001.zip › sensors-2309364-supplementary.pdf]

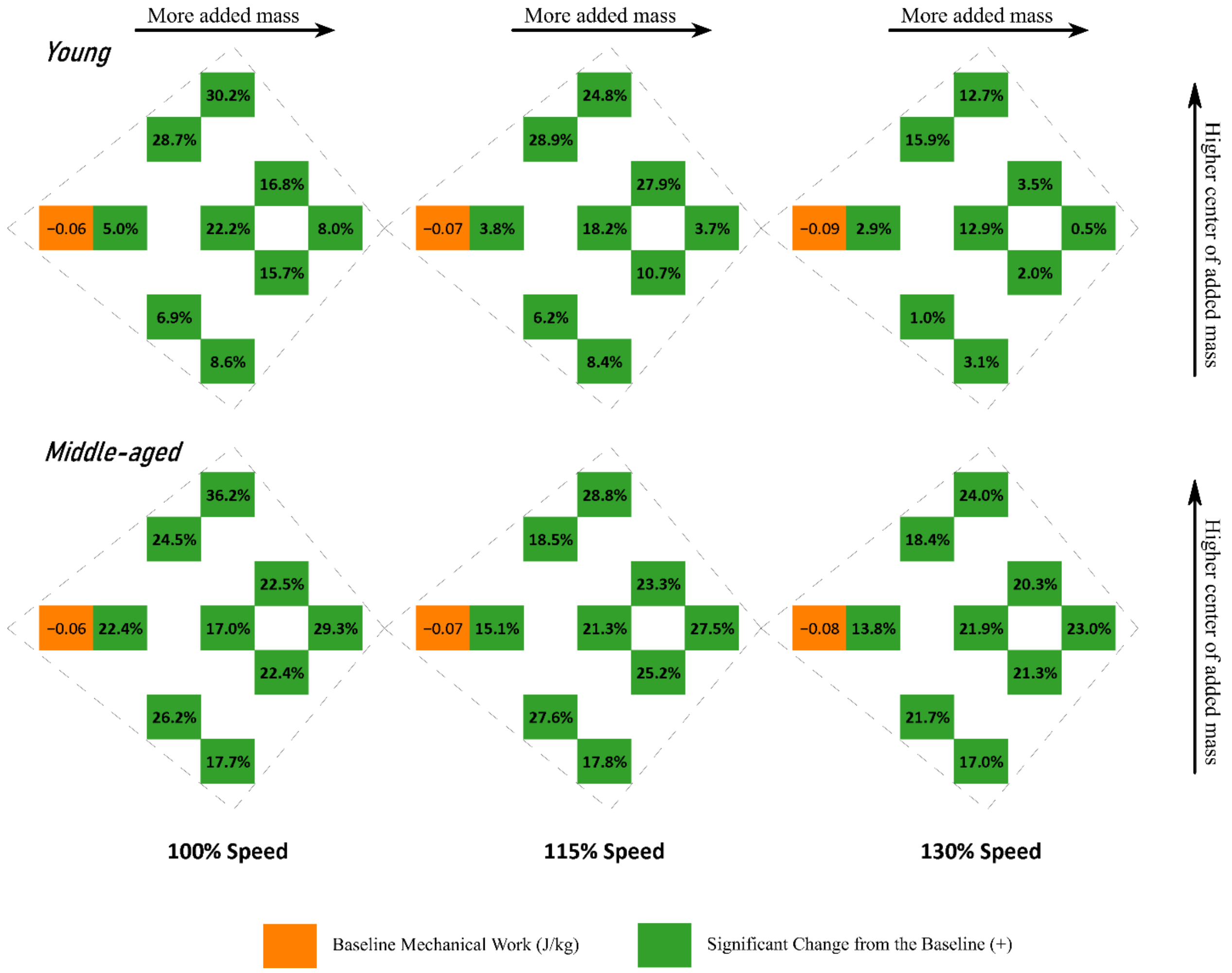

Figure S1. Changes in the sagittal plane hip joint mechanical work (W_{EX}) during hip extension. Results for young adults are located in the first row. Results for middle-aged adults are located in the second row. Results for walking at different speeds (100, 115, and 130%) are located in the columns. Baseline conditions are highlighted in orange with a unit of J/kg. Other values are relative differences with respect to the baseline value within the same cluster. Comparisons that are statistically significant ($p < 0.05$) are in bold with a green background for a positive (+) change. Within each cluster, added mass conditions with a higher total amount of added mass sit further to the right, and those with a higher center of added mass sit further away from the bottom.

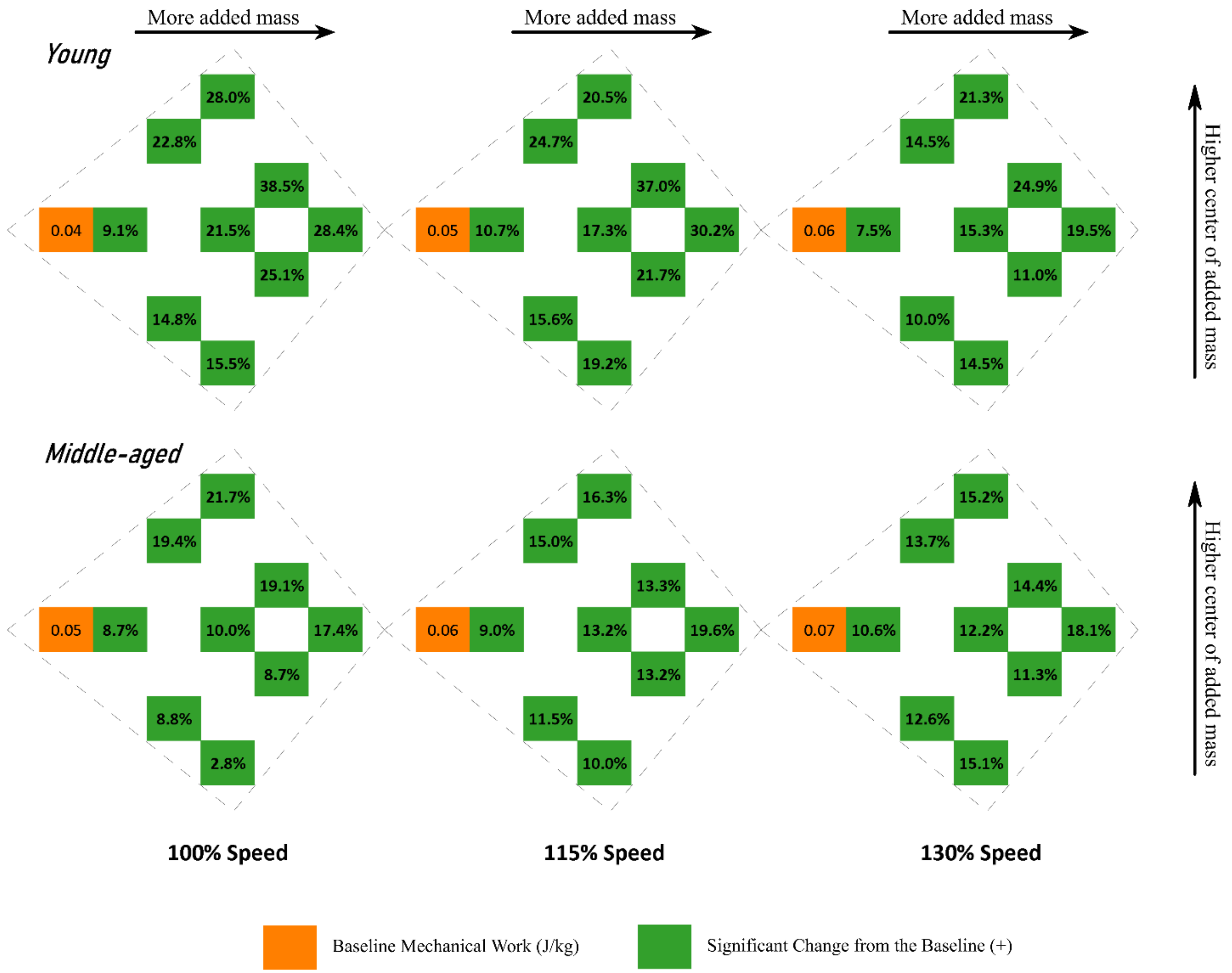

Figure S2. Changes in the sagittal plane hip joint mechanical work (W_{FL}) during hip flexion. Results for young adults are located in the first row. Results for middle-aged adults are located in the second row. Results for walking at different speeds (100, 115, and 130%) are located in the columns. Baseline conditions are highlighted in orange with a unit of J/kg. Other values are relative differences with respect to the baseline value within the same cluster. Comparisons that are statistically significant ($p < 0.05$) are in bold with a green background for a positive (+) change. Within each cluster, added mass conditions with a higher total amount of added mass sit further to the right, and those with a higher center of added mass sit further away from the bottom.

Table S1. Pairwise comparisons: Sagittal plane hip joint quasi-stiffness (K_{EX}) during hip extension.

	Speed	100%				115%				130%			
Young	Condition	*00*	*11*	*12*	*13*	*00*	*11*	*12*	*13*	*00*	*11*	*12*	*13*
	Mean	2.24	2.30	2.36	2.38	2.49	2.53	2.59	2.63	2.75	2.76	2.82	2.85
	SD	0.46	0.47	0.50	0.50	0.51	0.53	0.52	0.56	0.54	0.58	0.54	0.58
	p-value		**< 0.0001**	**< 0.0001**	**< 0.0001**		0.2603	**< 0.0001**	**< 0.0001**		**< 0.0001**	**< 0.0001**	**< 0.0001**
	Condition		*21*	*22*	*23*		*21*	*22*	*23*		*21*	*22*	*23*
	Mean		2.41	2.37	2.41		2.64	2.63	2.62		2.94	2.90	2.91
	SD		0.53	0.50	0.51		0.54	0.50	0.57		0.65	0.58	0.68
	p-value		**< 0.0001**	**< 0.0001**	**< 0.0001**		**< 0.0001**	**< 0.0001**	**< 0.0001**		0.2834	**< 0.0001**	**< 0.0001**
	Condition		*31*	*32*	*33*		*31*	*32*	*33*		*31*	*32*	*33*
	Mean		2.44	2.41	2.50		2.66	2.74	2.73		2.97	2.99	2.98
	SD		0.57	0.49	0.51		0.62	0.61	0.56		0.69	0.72	0.66
	p-value		**< 0.0001**	**< 0.0001**	**< 0.0001**		**< 0.0001**	**< 0.0001**	**< 0.0001**		**< 0.0001**	**< 0.0001**	**< 0.0001**
Middle-aged	Condition	*00*	*11*	*12*	*13*	*00*	*11*	*12*	*13*	*00*	*11*	*12*	*13*
	Mean	2.40	2.42	2.29	2.43	2.64	2.64	2.51	2.59	2.83	2.88	2.71	2.81
	SD	0.75	0.71	0.64	0.66	0.78	0.75	0.66	0.59	0.80	0.77	0.67	0.62
	p-value		**< 0.0001**	**< 0.0001**	**< 0.0001**		0.9941	**< 0.0001**	0.1408		**< 0.0001**	0.7887	**< 0.0001**
	Condition		*21*	*22*	*23*		*21*	*22*	*23*		*21*	*22*	*23*
	Mean		2.49	2.50	2.44		2.74	2.70	2.65		2.97	2.90	2.86
	SD		0.67	0.70	0.70		0.70	0.69	0.68		0.72	0.72	0.67
	p-value		**< 0.0001**	**< 0.0001**	**< 0.0001**		**< 0.0001**	**< 0.0001**	0.8930		**< 0.0001**	**< 0.0001**	**< 0.0001**
	Condition		*31*	*32*	*33*		*31*	*32*	*33*		*31*	*32*	*33*
	Mean		2.48	2.47	2.51		2.67	2.71	2.68		2.88	2.88	2.92
	SD		0.67	0.71	0.64		0.68	0.72	0.71		0.70	0.74	0.73
	p-value		**< 0.0001**	**< 0.0001**	**< 0.0001**		0.6567	**< 0.0001**	**< 0.0001**		**< 0.0001**	**< 0.0001**	**< 0.0001**

Mean and SD (Standard deviation) are in Nm/kg/rad. Bold p-values < 0.05. Comparisons are with respect to the baseline, "00" condition, within the same speed and age category. Condition names are defined in Table 1.

Table S2. Pairwise comparisons: Sagittal plane hip joint quasi-stiffness (K_{FL}) during hip flexion.

	Speed	100%				115%				130%			
Young	Condition	*00*	*11*	*12*	*13*	*00*	*11*	*12*	*13*	*00*	*11*	*12*	*13*
	Mean	2.42	2.20	2.21	2.26	2.82	2.51	2.52	2.65	3.40	2.96	2.95	3.05
	SD	0.66	0.58	0.60	0.63	0.75	0.66	0.63	0.66	0.94	0.74	0.82	0.79
	p-value	-	**< 0.0001**	**< 0.0001**	**< 0.0001**	-	**< 0.0001**	**< 0.0001**	**< 0.0001**	-	**< 0.0001**	**< 0.0001**	**< 0.0001**
	Condition		*21*	*22*	*23*		*21*	*22*	*23*		*21*	*22*	*23*
	Mean		2.16	2.20	2.17		2.55	2.50	2.47		2.93	2.93	3.02
	SD		0.68	0.63	0.62		0.74	0.68	0.75		0.89	0.80	1.07
	p-value		**< 0.0001**	**< 0.0001**	**< 0.0001**		**< 0.0001**	**< 0.0001**	**< 0.0001**		**< 0.0001**	**< 0.0001**	**< 0.0001**
	Condition		*31*	*32*	*33*		*31*	*32*	*33*		*31*	*32*	*33*
	Mean		2.19	2.19	2.25		2.49	2.46	2.48		2.86	2.84	2.88
	SD		0.69	0.62	0.70		0.79	0.60	0.90		0.84	0.82	0.99
	p-value		**< 0.0001**	**< 0.0001**	**< 0.0001**		**< 0.0001**	**< 0.0001**	**< 0.0001**		**< 0.0001**	**< 0.0001**	**< 0.0001**
Middle-aged	Condition	*00*	*11*	*12*	*13*	*00*	*11*	*12*	*13*	*00*	*11*	*12*	*13*
	Mean	2.92	2.86	2.89	2.92	3.30	3.25	3.32	3.35	3.78	3.71	3.71	3.78
	SD	1.11	0.94	0.94	1.02	1.21	1.06	1.05	1.11	1.33	1.21	1.20	1.20
	p-value		0.4786	0.9220	0.9948	-	0.7780	0.9224	0.6401	-	**< 0.0001**	0.0707	0.9980
	Condition		*21*	*22*	*23*		*21*	*22*	*23*		*21*	*22*	*23*
	Mean		2.86	2.78	2.98		3.19	3.19	3.40		3.66	3.66	3.92
	SD		0.95	0.93	1.08		1.01	1.05	1.18		1.15	1.16	1.34
	p-value		0.5404	**< 0.0001**	0.5951		**< 0.0001**	**< 0.0001**	**< 0.0001**		**< 0.0001**	**< 0.0001**	**< 0.0001**
	Condition		*31*	*32*	*33*		*31*	*32*	*33*		*31*	*32*	*33*
	Mean		2.86	2.83	2.92		3.16	3.28	3.31		3.61	3.67	3.79
	SD		0.97	0.94	0.93		1.00	1.07	1.01		1.14	1.20	1.18
	p-value		**< 0.0001**	**0.0020**	0.9981		**< 0.0001**	0.9407	**< 0.0001**		**< 0.0001**	**< 0.0001**	**< 0.0001**

Mean and SD (Standard deviation) are in Nm/kg/rad. Bold *p*-values < 0.05. Comparisons are with respect to the baseline, “00” condition, within the same speed and age category. Condition names are defined in Table 1.

Table S3. Pairwise comparisons: Sagittal plane hip joint mechanical work (W_{EX}) during hip extension.

Speed		100%				115%				130%			
Young	Condition	*00*	*11*	*12*	*13*	*00*	*11*	*12*	*13*	*00*	*11*	*12*	*13*
	Mean	-0.06	-0.06	-0.06	-0.06	-0.07	-0.07	-0.07	-0.08	-0.09	-0.09	-0.09	-0.09
	SD	0.03	0.03	0.03	0.03	0.03	0.03	0.03	0.03	0.03	0.04	0.04	0.04
	p-value		**< 0.0001**	**< 0.0001**	**< 0.0001**		0.4534	**0.0002**	**< 0.0001**		**< 0.0001**	**< 0.0001**	**< 0.0001**
	Condition		*21*	*22*	*23*		*21*	*22*	*23*		*21*	*22*	*23*
	Mean		-0.07	-0.07	-0.07		-0.09	-0.08	-0.08		-0.10	-0.10	-0.09
	SD		0.04	0.03	0.03		0.05	0.04	0.04		0.05	0.04	0.04
	p-value		**< 0.0001**	**< 0.0001**	**< 0.0001**		**< 0.0001**	**< 0.0001**	**< 0.0001**		**< 0.0001**	**< 0.0001**	**< 0.0001**
	Condition		*31*	*32*	*33*		*31*	*32*	*33*		*31*	*32*	*33*
	Mean		-0.07	-0.07	-0.06		-0.09	-0.09	-0.07		-0.10	-0.09	-0.09
	SD		0.05	0.03	0.03		0.05	0.08	0.04		0.05	0.04	0.04
	p-value		**< 0.0001**	**< 0.0001**	**< 0.0001**		**< 0.0001**	**< 0.0001**	**< 0.0001**		**< 0.0001**	**< 0.0001**	**< 0.0001**
Middle-aged	Condition	*00*	*11*	*12*	*13*	*00*	*11*	*12*	*13*	*00*	*11*	*12*	*13*
	Mean	-0.06	-0.07	-0.08	-0.07	-0.07	-0.08	-0.09	-0.08	-0.08	-0.09	-0.10	-0.10
	SD	0.07	0.08	0.08	0.08	0.07	0.08	0.08	0.08	0.07	0.08	0.08	0.08
	p-value		**< 0.0001**	**< 0.0001**	**< 0.0001**		**< 0.0001**	**< 0.0001**	**< 0.0001**		**< 0.0001**	**< 0.0001**	**< 0.0001**
	Condition		*21*	*22*	*23*		*21*	*22*	*23*		*21*	*22*	*23*
	Mean		-0.07	-0.07	-0.07		-0.08	-0.09	-0.09		-0.10	-0.10	-0.10
	SD		0.07	0.07	0.08		0.07	0.08	0.08		0.07	0.08	0.08
	p-value		**< 0.0001**	**< 0.0001**	**< 0.0001**		**< 0.0001**	**< 0.0001**	**< 0.0001**		**< 0.0001**	**< 0.0001**	**< 0.0001**
	Condition		*31*	*32*	*33*		*31*	*32*	*33*		*31*	*32*	*33*
	Mean		-0.08	-0.07	-0.08		-0.09	-0.09	-0.09		-0.10	-0.10	-0.10
	SD		0.08	0.07	0.08		0.07	0.07	0.08		0.07	0.08	0.07
	p-value		**< 0.0001**	**< 0.0001**	**< 0.0001**		**< 0.0001**	**< 0.0001**	**< 0.0001**		**< 0.0001**	**< 0.0001**	**< 0.0001**

Mean and SD (Standard deviation) are in J/kg. Bold *p*-values < 0.05. Comparisons are with respect to the baseline, “00” condition, within the same speed and age category. Condition names are defined in Table 1.

Table S4. Pairwise comparisons: Sagittal plane hip joint mechanical work (W_{FL}) during hip flexion.

	Speed	100%				115%				130%			
Young	Condition	*00*	*11*	*12*	*13*	*00*	*11*	*12*	*13*	*00*	*11*	*12*	*13*
	Mean	0.04	0.04	0.04	0.04	0.05	0.06	0.06	0.06	0.06	0.07	0.07	0.07
	SD	0.01	0.02	0.02	0.02	0.02	0.02	0.02	0.03	0.02	0.03	0.03	0.03
	p-value		**< 0.0001**	**< 0.0001**	**< 0.0001**		**< 0.0001**	**< 0.0001**	**< 0.0001**		**< 0.0001**	**< 0.0001**	**< 0.0001**
	Condition		*21*	*22*	*23*		*21*	*22*	*23*		*21*	*22*	*23*
	Mean		0.05	0.05	0.05		0.06	0.06	0.06		0.07	0.07	0.07
	SD		0.02	0.02	0.02		0.02	0.02	0.02		0.03	0.03	0.03
	p-value		**< 0.0001**	**< 0.0001**	**< 0.0001**		**< 0.0001**	**< 0.0001**	**< 0.0001**		**< 0.0001**	**< 0.0001**	**< 0.0001**
	Condition		*31*	*32*	*33*		*31*	*32*	*33*		*31*	*32*	*33*
	Mean		0.05	0.05	0.05		0.06	0.07	0.06		0.08	0.08	0.08
	SD		0.02	0.02	0.02		0.03	0.03	0.03		0.03	0.03	0.03
	p-value		**< 0.0001**	**< 0.0001**	**< 0.0001**		**< 0.0001**	**< 0.0001**	**< 0.0001**		**< 0.0001**	**< 0.0001**	**< 0.0001**
Middle-aged	Condition	*00*	*11*	*12*	*13*	*00*	*11*	*12*	*13*	*00*	*11*	*12*	*13*
	Mean	0.05	0.05	0.05	0.05	0.06	0.06	0.06	0.06	0.07	0.08	0.08	0.08
	SD	0.03	0.02	0.03	0.02	0.03	0.03	0.03	0.03	0.03	0.03	0.03	0.04
	p-value		**< 0.0001**	**< 0.0001**	0.7479		**< 0.0001**	**< 0.0001**	**< 0.0001**		**< 0.0001**	**< 0.0001**	**< 0.0001**
	Condition		*21*	*22*	*23*		*21*	*22*	*23*		*21*	*22*	*23*
	Mean		0.05	0.05	0.05		0.07	0.06	0.06		0.08	0.08	0.08
	SD		0.03	0.03	0.03		0.03	0.03	0.03		0.04	0.03	0.04
	p-value		**< 0.0001**	**< 0.0001**	**< 0.0001**		**< 0.0001**	**< 0.0001**	**< 0.0001**		**< 0.0001**	**< 0.0001**	**< 0.0001**
	Condition		*31*	*32*	*33*		*31*	*32*	*33*		*31*	*32*	*33*
	Mean		0.06	0.05	0.05		0.07	0.06	0.07		0.08	0.08	0.08
	SD		0.03	0.03	0.03		0.03	0.03	0.03		0.04	0.03	0.04
	p-value		**< 0.0001**	**< 0.0001**	**< 0.0001**		**< 0.0001**	**< 0.0001**	**< 0.0001**		**< 0.0001**	**< 0.0001**	**< 0.0001**

Mean and SD (Standard deviation) are in J/kg. Bold *p*-values < 0.05. Comparisons are with respect to the baseline, “00” condition, within the same speed and age category. Condition names are defined in Table 1.

Table S5. Sagittal plane hip joint quasi-stiffness (K_{EX}) for each participant during hip extension.

Participant	Gender	Age	Age category	Body mass	Body height	#K_{EX}	Mean R^2	Mean RMSE	#Frames	K_{EX} range	Mean W_{EX}
1	M	22	Y	76.35	1.73	3144	0.93	0.074	36	[1.560, 4.620]	-0.101
2	M	23	Y	67.30	1.75	3064	0.96	0.044	32	[1.324, 3.229]	-0.059
3	F	23	Y	68.71	1.78	2224	0.95	0.039	35	[1.077, 6.437]	-0.093
4	M	20	Y	56.71	1.78	2697	0.88	0.122	41	[1.586, 4.095]	-0.074
5	F	53	M	74.84	1.65	3040	0.96	0.044	31	[1.380, 4.230]	-0.072
6	F	23	Y	79.66	1.63	3530	0.93	0.064	29	[1.309, 5.703]	-0.080
7	F	37	M	76.45	1.56	3350	0.90	0.077	43	[1.457, 3.094]	-0.091
8	M	31	Y	68.94	1.83	3120	0.97	0.049	33	[2.540, 4.977]	-0.064
9	F	36	M	68.94	1.63	3368	0.95	0.054	39	[1.528, 4.026]	-0.102
10	M	41	M	76.37	1.82	3340	0.88	0.117	34	[2.884, 5.423]	-0.061
11	F	36	M	85.33	1.71	3121	0.81	0.144	46	[1.164, 3.383]	-0.081
12	F	41	M	71.78	1.70	3144	0.88	0.077	38	[1.402, 2.939]	-0.046
13	M	54	M	102.84	1.83	2413	0.92	0.089	51	[1.415, 2.832]	-0.134
14	M	40	M	89.48	1.71	3270	0.90	0.090	34	[2.388, 5.779]	-0.054
15	F	20	Y	62.22	1.65	2194	0.92	0.058	47	[1.291, 2.877]	-0.034
16	F	18	Y	67.08	1.71	2453	0.96	0.053	36	[1.695, 3.946]	-0.092
17	M	19	Y	73.80	1.80	2614	0.96	0.036	29	[1.899, 4.662]	-0.054
18	M	18	Y	67.81	1.69	3063	0.94	0.069	44	[1.351, 3.227]	-0.135
19	F	22	Y	70.31	1.63	3057	0.97	0.029	27	[1.682, 4.094]	-0.030
20	F	38	M	53.77	1.68	2808	0.93	0.071	41	[1.067, 3.088]	-0.047
21	F	37	M	54.84	1.59	3371	0.90	0.091	31	[1.580, 5.197]	-0.098
22	F	38	M	59.01	1.68	2935	0.95	0.051	34	[1.704, 4.086]	-0.042
23	F	43	M	64.09	1.73	2748	0.92	0.109	46	[1.745, 3.810]	-0.356
24	M	60	M	79.48	1.83	2317	0.96	0.031	25	[1.047, 5.580]	-0.038
25	M	27	Y	64.09	1.80	2908	0.93	0.078	46	[1.332, 3.100]	-0.129
26	F	44	M	56.20	1.57	2520	0.97	0.024	35	[0.873, 3.583]	-0.021
27	M	61	M	92.28	1.82	2995	0.97	0.026	20	[1.603, 6.040]	-0.029
28	M	55	M	79.62	1.71	2945	0.95	0.068	34	[1.893, 5.141]	-0.112
29	M	27	Y	88.67	1.77	3083	0.92	0.064	38	[1.646, 3.827]	-0.065
			**Mean**	**72.83**	**1.71**	**2820**	**0.93**	**0.068**	**36**	**2.651**	**-0.082**
			**SD**	**12.86**	**0.08**	**509**	**0.05**	**0.037**	**8**	**0.675**	**0.063**

Body mass in kilograms;
Body height in meters;
Age category: Y for young adults, and M for middle-aged adults;
#K_{EX}: Number of hip joint quasi-stiffness values during hip extension, both sides included;
Mean R^2: Mean R-squared value of the curve fitting;
Mean RMSE: Mean root mean squared error of the curve fitting (Nm/kg);
#Frames: Average number of frames used for the curve fitting for each K_{EX} value;
K_{EX} range: Range of K_{EX} (Nm/kg/rad);
Mean W_{EX}: Average mechanical work at the hip joint during hip extension (J/kg);
SD: Standard deviation;
Sampling frequency: 150 Hz.

Table S6. Sagittal plane hip joint quasi-stiffness (K_{FL}) for each participant during hip flexion.

Participant	Gender	Age	Age category	Body mass	Body height	#K_{FL}	Mean R^2	Mean RMSE	#Frames	K_{FL} range	Mean W_{FL}
1	M	22	Y	76.35	1.73	3144	0.89	0.106	16	[1.516, 8.060]	0.050
2	M	23	Y	67.30	1.75	3064	0.91	0.069	19	[0.612, 3.974]	0.054
3	F	23	Y	68.71	1.78	2224	0.88	0.075	25	[0.388, 4.718]	0.066
4	M	20	Y	56.71	1.78	2697	0.80	0.133	21	[0.950, 4.334]	0.091
5	F	53	M	74.84	1.65	3040	0.93	0.071	18	[1.009, 5.211]	0.064
6	F	23	Y	79.66	1.63	3530	0.91	0.071	17	[1.251, 6.082]	0.076
7	F	37	M	76.45	1.56	3350	0.94	0.074	17	[1.829, 4.371]	0.058
8	M	31	Y	68.94	1.83	3120	0.86	0.094	22	[0.654, 4.098]	0.081
9	F	36	M	68.94	1.63	3368	0.94	0.079	18	[2.092, 6.288]	0.072
10	M	41	M	76.37	1.82	3340	0.93	0.101	20	[3.291, 7.604]	0.064
11	F	36	M	85.33	1.71	3121	0.96	0.070	20	[1.627, 4.172]	0.104
12	F	41	M	71.78	1.70	3144	0.96	0.053	18	[2.069, 6.303]	0.040
13	M	54	M	102.84	1.83	2413	0.83	0.137	19	[1.675, 6.910]	0.050
14	M	40	M	89.48	1.71	3270	0.86	0.094	17	[1.583, 8.252]	0.046
15	F	20	Y	62.22	1.65	2194	0.89	0.066	23	[1.026, 2.731]	0.045
16	F	18	Y	67.08	1.71	2453	0.95	0.062	21	[1.534, 3.921]	0.100
17	M	19	Y	73.80	1.80	2614	0.94	0.060	20	[1.532, 4.186]	0.044
18	M	18	Y	67.81	1.69	3063	0.84	0.129	22	[1.402, 5.513]	0.060
19	F	22	Y	70.31	1.63	3057	0.76	0.092	20	[0.679, 3.506]	0.024
20	F	38	M	53.77	1.68	2808	0.82	0.118	21	[1.038, 9.157]	0.063
21	F	37	M	54.84	1.59	3371	0.96	0.065	19	[1.764, 5.019]	0.093
22	F	38	M	59.01	1.68	2935	0.89	0.092	18	[0.982, 7.018]	0.036
23	F	43	M	64.09	1.73	2748	0.93	0.153	20	[3.589, 7.387]	0.109
24	M	60	M	79.48	1.83	2318	0.95	0.058	21	[0.576, 4.300]	0.056
25	M	27	Y	64.09	1.80	2908	0.88	0.128	21	[1.951, 6.715]	0.040
26	F	44	M	56.20	1.57	2520	0.85	0.058	22	[0.560, 2.773]	0.014
27	M	61	M	92.28	1.82	2999	0.95	0.038	15	[1.419, 5.281]	0.026
28	M	55	M	79.62	1.71	2945	0.96	0.068	17	[1.978, 6.334]	0.117
29	M	27	Y	88.67	1.77	3083	0.87	0.099	21	[1.436, 5.582]	0.044
			**Mean**	**72.83**	**1.71**	**2884**	**0.90**	**0.086**	**20**	**2.999**	**0.062**
			**SD**	**12.86**	**0.08**	**388**	**0.07**	**0.036**	**3**	**1.083**	**0.031**

Body mass in kilograms;
Body height in meters;
Age category: Y for young adults, and M for middle-aged adults;
#K_{FL}: Number of hip joint quasi-stiffness values during hip flexion, both sides included;
Mean R^2: Mean R-squared value of the curve fitting;
Mean RMSE: Mean root mean squared error of the curve fitting (Nm/kg);
#Frames: Average number of frames used for the curve fitting for each K_{FL} value;
K_{FL} range: Range of K_{FL} (Nm/kg/rad);
Mean W_{FL}: Average mechanical work at the hip joint during hip flexion (J/kg);
SD: Standard deviation;
Sampling frequency: 150 Hz
